# A Powerful Approach to Sub-Phenotype Analysis in Population-Based Genetic Association Studies

**DOI:** 10.1002/gepi.20486

**Published:** 2009-12-28

**Authors:** Andrew P Morris, Cecilia M Lindgren, Eleftheria Zeggini, Nicholas J Timpson, Timothy M Frayling, Andrew T Hattersley, Mark I McCarthy

**Affiliations:** 1 The Wellcome Trust Centre for Human Genetics, University of OxfordOxford, United Kingdom; 2 The Wellcome Trust Sanger InstituteHinxton, Cambridge, United Kingdom; 3 Medical Research Council Centre for Causal Analyses in Transitional Epidemiology, University of BristolUnited Kingdom; 4 Genetics of Complex Traits, Institute of Biomedical and Clinical Science, Peninsula Medical SchoolExeter, United Kingdom; 5 Diabetes Genetics, Institute of Biomedical and Clinical Science, Peninsula Medical SchoolExeter, United Kingdom; 6 Oxford Centre for Diabetes, Endocrinology and Metabolism, University of OxfordOxford, United Kingdom

**Keywords:** multinomial regression, sub-phenotype analysis, genome-wide association study, type 2 diabetes, obesity

## Abstract

The ultimate goal of genome-wide association (GWA) studies is to identify genetic variants contributing effects to complex phenotypes in order to improve our understanding of the biological architecture underlying the trait. One approach to allow us to meet this challenge is to consider more refined sub-phenotypes of disease, defined by pattern of symptoms, for example, which may be physiologically distinct, and thus may have different underlying genetic causes. The disadvantage of sub-phenotype analysis is that large disease cohorts are sub-divided into smaller case categories, thus reducing power to detect association. To address this issue, we have developed a novel test of association within a multinomial regression modeling framework, allowing for heterogeneity of genetic effects between sub-phenotypes. The modeling framework is extremely flexible, and can be generalized to any number of distinct sub-phenotypes. Simulations demonstrate the power of the multinomial regression-based analysis over existing methods when genetic effects differ between sub-phenotypes, with minimal loss of power when these effects are homogenous for the unified phenotype. Application of the multinomial regression analysis to a genome-wide association study of type 2 diabetes, with cases categorized according to body mass index, highlights previously recognized differential mechanisms underlying obese and non-obese forms of the disease, and provides evidence of a potential novel association that warrants follow-up in independent replication cohorts.

## INTRODUCTION

Genome-wide association (GWA) studies, such as those undertaken by the Wellcome Trust Case Control Consortium (WTCCC) [The Wellcome Trust Case Control Consortium, 2007], have proved to be extremely successful in identifying novel genetic components underlying complex human disease. Much of this success is due to better understanding of common human genetic variation [The International HapMap Consortium, 2007], improvements in the throughput and cost-efficiency of genome-wide genotyping platforms, and the availability of large, well characterized, population-based cohorts that provide sufficient power to detect the modest effects we expect for complex traits. Large international consortia are now undertaking collaborative meta-analyses of the results of GWA studies across populations with common ancestry, utilizing effective sample sizes of tens of thousands of individuals for discovery and replication of increasingly modest genetic effects contributing to traits such as type 2 diabetes (T2D) [Zeggini et al., 2008], Crohn's disease (CD) [Barrett et al., 2008], obesity [Willer et al., 2009], rheumatoid arthritis [Raychaudhuri et al., 2008], and schizophrenia [O'Donovan et al., 2008]. However, despite these successes, much of the genetic contributions to these, and other complex traits, remain unexplained.

One approach to advance our understanding of the biological mechanisms underlying a phenotype under investigation is to refine the trait, somehow. These *sub-phenotypes* could be defined by severity of disease, age of onset, or the site and/or pattern of symptoms, such as we see in inflammatory bowel disease, for example. By doing this, we may detect associations with variants contributing different effects to sub-phenotypes that would otherwise be overlooked by considering all cases, simultaneously, as the same phenotype. However, by focusing on specific sub-phenotypes, we reduce sample size, and thus will lose power to map loci contributing homogeneous effects to the unified phenotype.

In order to address this issue, we have developed a novel test for disease association, allowing for heterogeneity in genetic effects between sub-phenotypes, within a multinomial regression framework. We demonstrate, by simulation, that the multinomial regression approach has greater power to detect disease association, in the presence of heterogeneity in allelic odds ratios between sub-phenotypes, than do existing methods formulated in a logistic regression framework. Furthermore, when genetic effects are consistent across sub-phenotypes, the loss in power of the multinomial regression analysis is minimal, despite the additional parameters required in the model.

To demonstrate the utility of our multinomial regression approach, we have re-analyzed a GWA study of T2D from the main WTCCC experiment [The Wellcome Trust Case Control Consortium, 2007] by categorizing cases according to obesity, a well established risk factor for the disease, typically assessed by body mass index (BMI). The clear relationship between T2D and obesity would suggest that variants associated with BMI may also influence susceptibility to the disease. For example, analysis of the main WTCCC experiment highlighted strong evidence of association of T2D with variants in *FTO* (trend test *P* 5 5.2 × 10^−8^). However, analysis of BMI as a continuous trait in the aforementioned case samples demonstrated strong evidence of obesity association with precisely the same variants (trend test *P* = 8.0 × 10^−6^). In particular, high-risk alleles for T2D were also associated with increased BMI [Frayling et al., 2007]. Our multinomial regression analysis of the GWA study, allowing for heterogeneity of genetic effects between obese and non-obese cases, provides stronger signals of association at several of the now established T2D loci than do conventional logistic regression-based methods applied to all cases combined. Our results confirm previous findings of heterogeneity in genetic effects according to obesity sub-phenotype at variants in *FTO* and *TCF7L2* [Cauchi et al., 2006, 2008; Freathy et al., 2008; Timpson et al., 2009], and highlight a potential novel T2D association that warrants follow-up in replication cohorts.

## MODEL AND METHODS

### MODEL FORMULATION AND ANALYSIS FRAMEWORK

Consider a case-control sample of unrelated individuals, where cases are categorized according to *K* possible disjoint sub-phenotypes. We denote the phenotype of the *i*th individual by *y_i_*, where *y_i_* = 0 for controls, and *y_i_* = *k* for cases with the *k*th sub-phenotype. Under the assumption of a linear trend in the allelic odds ratio (i.e. multiplicative disease risks), we can model the log-odds of the *k*th sub-phenotype for the *i*th individual in a multinomial regression framework, given by


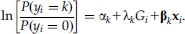
(1)

In this expression, *G_i_* denotes the SNP genotype of the *i*th individual, coded as 0,1, or 2, according to the number of minor alleles they carry. Furthermore, *x_i_* denotes a vector of their covariate measurements, with corresponding regression coefficients β_*k*_. The parameter λ_*k*_ represents the allelic log-odds ratio for the minor allele, relative to the major allele, for the *k*th sub-phenotype.

Within a multinomial regression framework, the log-likelihood contribution of the *i*th individual is given by


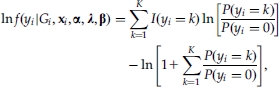


where *I(y_i_ = k)* is an indicator variable, taking the value 1 if they have the *k*th sub-phenotype, and 0 otherwise. We can then construct a likelihood ratio test of association of the SNP with disease, allowing for heterogeneity of allelic odds ratios between sub-phenotypes, by comparing the deviance of a model in which λ_*k*_ = 0 for all sub-phenotypes to that in which λ_*k*_ is unconstrained, given by


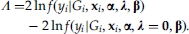


Under the null hypothesis of no association between the disease and SNP, Λ has an approximate χ^2^ distribution with *K* degrees of freedom.

Within a multinomial regression framework, we can also construct a test of heterogeneity of allelic odds ratios at the SNP between sub-phenotypes by comparing the deviance of a model in which λ_*k*_ = θ for all sub-phenotypes to that in which λ_*k*_ is unconstrained, given by


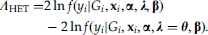


Under the null hypothesis of no heterogeneity of allelic odds ratios at the SNP between sub-phenotypes, Λ_HET_ has an approximate χ^2^ distribution with *K*−1 degrees of freedom.

The multinomial logistic regression framework described above is extremely flexible and can be easily extended to allow for non-multiplicative disease risks, for example, by including an additional indicator *I*(*G_i_* = 1) of dominance in equation (1). Furthermore, we can test for association with imputed genotype data within this framework by replacing *G_i_* in equation (1) with the expected genotype from the posterior distribution of calls [Marchini et al., 2007]. The multinomial regression model can be fitted using the *mlogit* function in R [R Development Core Team, 2009].

### SIMULATION STUDY

We have performed simulations to investigate the power of the multinomial regression framework to test for disease association and heterogeneity in allelic odds ratios between sub-phenotypes, and to compare its performance to existing logistic regression-based approaches. We considered a disease for which cases are categorized according to two sub-phenotypes, and examined a wide range of association scenarios, parameterized in terms of: (i) the minor allele frequency (MAF) of the causal SNP; and (ii) the heterozygous log-relative risk, under a multiplicative disease model, for each sub-phenotype.

For each scenario, we simulated 10,000 replicates of data, each consisting of causal SNP genotype data under the assumption of Hardy–Weinberg equilibrium (HWE) for 2,000 controls, 1,000 cases of sub-phenotype 1 and 1,000 cases of sub-phenotype 2. For each replicate of data, we performed the following tests of association and heterogeneity, and recorded the P-value for each.

1. MULTINOMIAL: test of association of the causal SNP with disease, allowing heterogeneity of allelic odds ratios between sub-phenotypes, within a multinomial regression framework (2,000 cases against 2,000 controls).
2. LOGISTIC: test of association of the causal SNP with disease, assuming the genetic effect to be the same for both sub-phenotypes, within a logistic regression framework (2,000 cases against 2,000 controls).
3. SP1 and SP2: tests of association of the causal SNP with each sub-phenotype, separately, within a logistic regression framework (1,000 cases each against 2,000 shared controls).
4. HETEROGENEITY: test of heterogeneity of the effect of the causal SNP between sub-phenotypes within a multinomial regression framework (2,000 cases against 2,000 controls).
5. SP1vSP2: test of heterogeneity of the effect of the causal SNP between sub-phenotypes within a logistic regression framework (1,000 cases of sub- phenotype 1 against 1,000 cases of sub-phenotype 2).

For each test, we estimate power by the proportion of replicates for which the P-value meets a nominal significance threshold of 5%.

### APPLICATION TO A GWA STUDY OF T2D OBESITY SUB-PHENOTYPES

The T2D component of the main WTCCC experiment [The Wellcome Trust Case Control Consortium, 2007] consists of 1,999 cases from the Diabetes UK Warren 2 repository, and 3,004 controls from the 1958 British Birth Cohort (58C) and the UK National Blood Service (NBS). All samples were genotyped using the Affymetrix GeneChip 500K Mapping Array Set that incorporates 500,568 SNPs, genome-wide. We utilized exactly the same quality control (QC) filters employed by the WTCCC to exclude samples and SNPs, full details of which are presented in the description of the main experiment [The Wellcome Trust Case Control Consortium, 2007]. Briefly, case and control samples were excluded on the basis of call rate, outlying genome-wide heterozygosity, discrepancies in WTCCC and external identifying information, non-Caucasian ancestry, duplication and apparent relatedness. SNPs were excluded on the basis of call rate, extreme deviation from HWE, differential allele and/or genotype frequencies between the 58C and NBS control cohorts, or manual visual inspection of genotype calls.

For our analysis, each T2D case was assigned to one of two obesity sub-phenotypes: non-obese (BMI ≤ 30 kg m^−2^) and obese (BMI >30 kg m^−2^). Cases passing QC filters, but with unknown BMI, were unclassified and hence excluded from the analysis. For each SNP passing QC filters, the following tests were performed:

1. disease association within a multinomial regression framework (i.e. controls against obese and non-obese T2D sub-phenotypes);
2. disease association within a logistic regression framework (i.e. controls against obese and non-obese T2D cases combined);
3. heterogeneity of effects between obesity sub-pheno-types within a multinomial regression framework.

## RESULTS

### SIMULATION STUDY

Table I presents a summary of false-positive error rates, at a nominal significance level of 5%, of each multinomial or logistic regression-based test of association or heterogeneity. Estimates are based on 10,000 replicates of data generated under the null hypothesis of no association of the SNP with either phenotype, and are entirely consistent with the nominal significance level.

**Fig. 1:**
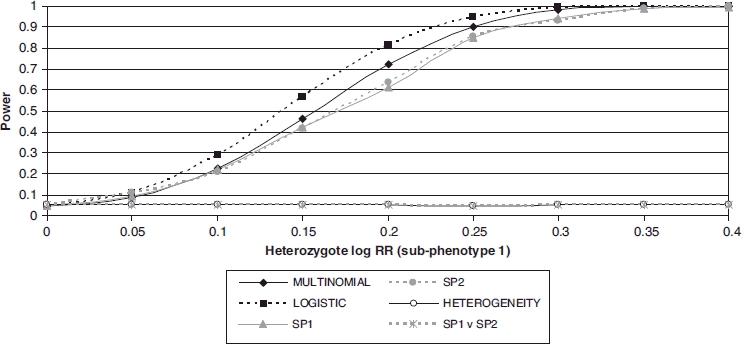
Power of tests of disease association and heterogeneity of genetic effects between two sub-phenotypes, where the causal variant (MAF10%) has the same effect on both sub-phenotypes. Results are presented as a function of the heterozygote log-relative risk at a 5% significance level. MULTINOMIAL: test of association of the causal variant with disease, allowing heterogeneity of allelic odds ratios between sub-phenotypes, within a multinomial regression framework (2,000 cases against 2,000 controls). LOGISTIC: test of association of the causal variant with disease, assuming the genetic effect to be the same for both sub-phenotypes, within a logistic regression framework (2,000 cases against 2,000 controls). SP1: test of association of the causal variant with disease sub-phenotype 1 within a logistic regression framework (1,000 cases against 2,000 controls). SP2: test of association of the causal variant with disease sub-phenotype 2 within a logistic regression framework (1,000 cases against 2,000 controls). HETEROGENEITY: test of heterogeneity of the effect of the causal variant between sub-phenotypes within a multinomial regression framework (2,000 cases against 2,000 controls). SP1vSP2: test of heterogeneity of the effect of the causal variant between sub-phenotypes within a logistic regression framework (1,000 cases of sub-phenotype 1 against 1,000 cases of sub-phenotype 2).

**Fig. 2:**
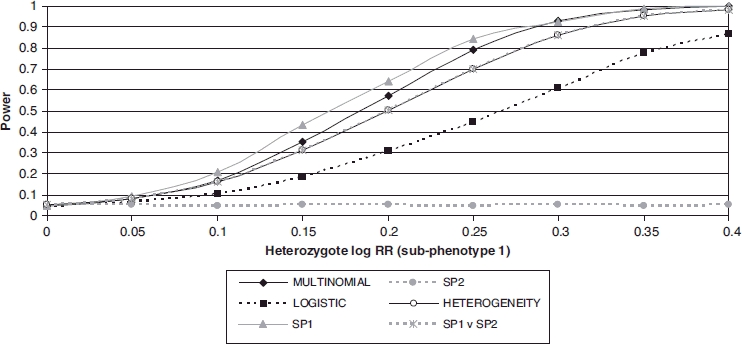
Power of tests of disease association and heterogeneity of genetic effects between two sub-phenotypes, where the causal variant (MAF10%) has no effect on sub-phenotype 2. Results are presented as a function of the heterozygote log-relative risk of sub-phenotype 1 at a 5% significance level. MULTINOMIAL: test of association of the causal variant with disease, allowing heterogeneity of allelic odds ratios between sub-phenotypes, within a multinomial regression framework (2,000 cases against 2,000 controls). LOGISTIC: test of association of the causal variant with disease, assuming the genetic effect to be the same for both sub-phenotypes, within a logistic regression framework (2,000 cases against 2,000 controls). SP1: test of association of the causal variant with disease sub-phenotype 1 within a logistic regression framework (1,000 cases against 2,000 controls). SP2: test of association of the causal variant with disease sub-phenotype 2 within a logistic regression framework (1,000 cases against 2,000 controls). HETEROGENEITY: test of heterogeneity of the effect of the causal variant between sub-phenotypes within a multinomial regression framework (2,000 cases against 2,000 controls). SP1vSP2: test of heterogeneity of the effect of the causal variant between sub-phenotypes within a logistic regression framework (1,000 cases of sub-phenotype 1 against 1,000 cases of sub-phenotype 2).

**Fig. 3:**
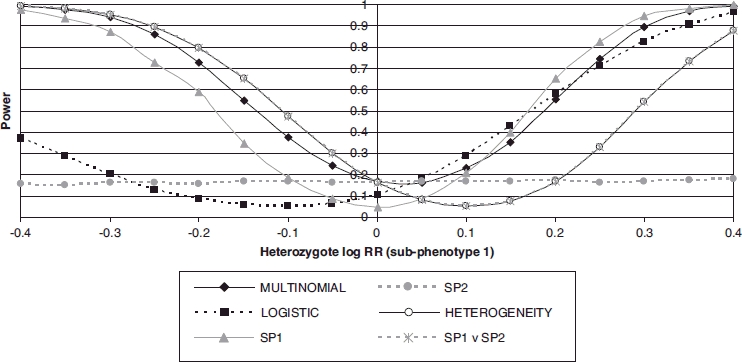
Power of tests of disease association and heterogeneity of genetic effects between two sub-phenotypes, where the causal variant (MAF 10%) has a fixed effect on sub-phenotype 2. Results are presented as a function of the heterozygote log-relative risk of sub-phenotype 1 at a 5% significance level, where the causal variant has a heterozygote log-relative risk of 0.1 for sub-phenotype 2. MULTINOMIAL: test of association of the causal variant with disease, allowing heterogeneity of allelic odds ratios between sub-phenotypes, within a multinomial regression framework (2,000 cases against 2,000 controls). LOGISTIC: test of association of the causal variant with disease, assuming the genetic effect to be the same for both sub-phenotypes, within a logistic regression framework (2,000 cases against 2,000 controls). SP1: test of association of the causal variant with disease sub-phenotype 1 within a logistic regression framework (1,000 cases against 2,000 controls). SP2: test of association of the causal variant with disease sub-phenotype 2 within a logistic regression framework (1,000 cases against 2,000 controls). HETEROGENEITY: test of heterogeneity of the effect of the causal variant between sub-phenotypes within a multinomial regression framework (2,000 cases against 2,000 controls). SP1vSP2: test of heterogeneity of the effect of the causal variant between sub-phenotypes within a logistic regression framework (1,000 cases of sub-phenotype 1 against 1,000 cases of sub-phenotype 2).

Figures 1–3 present the power, at a nominal significance level of 5%, of each multinomial or logistic regression-based test of association or heterogeneity, as a function of the heterozygote log-relative risk of disease for sub-phenotype 1. Results are presented for a causal variant with 10% MAF in three distinct settings: (i) the effect of the causal variant is the same for both sub-phenotypes (Fig. 1); (ii) the causal SNP has no effect on the sub-phenotype 2 (Fig. 2); and (iii) the causal SNP has a fixed heterozygote log-relative risk of disease of 0.1 for sub-phenotype 2 (Fig. 3).

**Table I:** False-positive error rates of tests of disease association and heterogeneity of genetic effects between two sub-phenotypes at a nominal 5% significance level

Test	Framework	Sample size	False-positive error rate % (standard error)
MULTINOMIAL	Multinomial	2,000 cases v 2,000 controls	4.72 (0.21)
LOGISTIC	Logistic	2,000 cases v 2,000 controls	4.72 (0.21)
SP1	Logistic	1,000 cases v 2,000 controls	4.70 (0.21)
SP2	Logistic	1,000 cases v 2,000 controls	5.19 (0.22)
HETEROGENEITY	Multinomial	2,000 cases v 2,000 controls	5.11 (0.22)
SP1vSP2	Logistic	1,000 cases v 1,000 cases	5.07 (0.21)

MULTINOMIAL: test of association of the causal variant with disease, allowing heterogeneity of allelic odds ratios between sub-phenotypes, within a multinomial regression framework (2,000 cases against 2,000 controls). LOGISTIC: test of association of the causal variant with disease, assuming the genetic effect to be the same for both sub-phenotypes, within a logistic regression framework (2,000 cases against 2,000 controls). SP1: test of association of the causal variant with disease sub-phenotype 1 within a logistic regression framework (1,000 cases against 2,000 controls). SP2: test of association of the causal variant with disease sub-phenotype 2 within a logistic regression framework (1,000 cases against 2,000 controls). HETEROGENEITY: test of heterogeneity of the effect of the causal variant between sub-phenotypes within a multinomial regression framework (2,000 cases against 2,000 controls). SP1vSP2: test of heterogeneity of the effect of the causal variant between sub-phenotypes within a logistic regression framework (1,000 cases of sub-phenotype 1 against 1,000 cases of sub-phenotype 2).

Figure 1 demonstrates that, in the scenario where the effect of the causal variant is the same for both sub-phenotypes, the multinomial regression analysis of cases categorized according to sub-phenotype is less powerful than conventional logistic regression analysis of all cases combined, although the difference is minimal (MULTINOMIAL compared with LOGISTIC). This is entirely expected since the additional parameter required to allow for heterogeneity in the multinomial regression model is unnecessary when the effect of the causal variant is the same for both sub-phenotypes. Encouragingly, the multinomial regression analysis is more powerful than logistic regression analysis of cases of each sub-phenotype, separately, against a shared cohort of controls (MULTINOMIAL compared with SP1 and SP2). In this setting, there is a trade-off of the additional parameter required in the multinomial regression model against the increased number of cases incorporated in the analysis.

Figure 2 illustrates that, in the scenario where the causal variant has an effect on only one sub-phenotype, logistic regression analysis of cases of the specific sub-phenotype against controls is more powerful than multinomial regression analysis of cases categorized according to sub-phenotype, although the difference is minimal (MULTINOMIAL compared with SP1). However, the multinomial regression model, which allows for heterogeneity of allelic effects between sub-phenotypes, has noticeably greater power than logistic regression analysis of all cases combined (MULTINOMIAL compared with LOGISTIC). Figure 3 demonstrates that multinomial regression analysis of cases categorized according to sub-phenotype performs well, compared with all other approaches, over a wide range of models in which the causal variant contributes effects to both sub-phenotypes, but not necessarily in the same direction (MULTINOMIAL compared with LOGISTIC, SP1 and SP2). Again, these results are expected since the multinomial regression model has been developed to allow for heterogeneity of effects between sub-phenotypes. The power of each of the two tests of heterogeneity is indistinguishable (HETEROGENEITY compared with SP1vSP2). Again, this is not unexpected since the controls do not contribute to our proposed test of heterogeneity derived within the multinomial regression framework. Simulations were also performed over a range of MAFs for the causal variant between 1 and 50%. Although the absolute power of each test varied dramatically over this interval, their relative performance remained consistent with our conclusions for 10% MAF (results not presented).

### APPLICATION TO T2D OBESITY SUB-PHENOTYPES

**Fig. 4:**
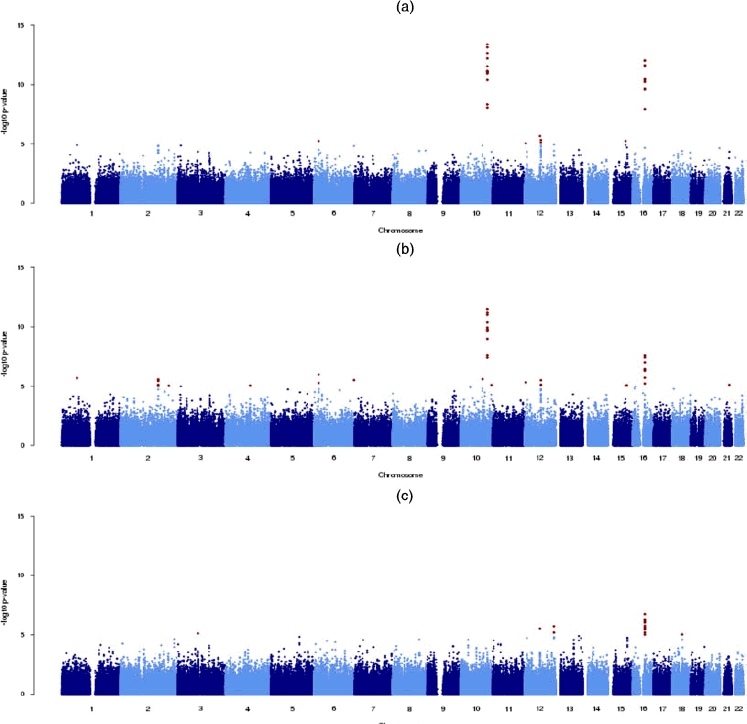
Manhattan plots to summarize results of a GWA study of 1,913 T2D cases and 2,938 controls: (a) multinomial regression analysis with cases categorized according to obesity sub-phenotypes; (b) logistic regression analysis with all cases combined; and (c) heterogeneity of effects between obesity sub-phenotypes. Results are presented for 393,143 autosomal SNPs passing QC filters with MAF > 1% across the complete case-control cohort. The strongest signals of association and heterogeneity (*P* < 10^−5^) are indicated in red.

A total of 4,851 samples from the T2D component of the main WTCCC experiment [The Wellcome Trust Case Control Consortium, 2007] passed QC filters: 2,938 controls and 1,924 cases. An additional 11 cases were excluded from the analysis due to unknown BMI. The median BMI among case samples is 30.3 kg m^−2^, leading to similar frequencies of obese (997) and non-obese (916) individuals when categorized according to the traditional obesity threshold (BMI >30). Figure 4 presents Manhattan plots for 393,143 autosomal SNPs passing QC filters with MAF >1% across the complete case-control cohort for tests of association with T2D, and heterogeneity of genetic effects according to obesity. The multinomial (Fig. 4a) and logistic (Fig. 4b) regression analyses produce similar results, in general, highlighting the same regions on chromosome 10 and 16 with the strongest evidence of association with T2D. The difference in the magnitude of signals, for example on chromosome 16, can be explained by the heterogeneity in allelic odds ratios between obese and non-obese T2D cases (Fig. 4c).

Table II presents a summary of regions of the genome demonstrating evidence of association with T2D (*P*<10^−5^) in a multinomial regression framework, with cases categorized according to obesity. The strongest signal of association was observed for variants in *TCF7L2* (lead SNP rs4506565), with more convincing evidence obtained from the multinomial regression analysis (*P* = 4.0 × 10^−14^) than the logistic regression analysis of all T2D cases, combined (*P* = 3.0 × 10^−12^). There is clear evidence of heterogeneity in allelic odds ratios between obesity categories (*P* = 3.0 × 10^−4^). Our results confirm previous findings that variants in *TCF7L2* have stronger effects on non-obese T2D cases than those that are obese [Cauchi et al., 2006, 2008; Timpson et al., 2009]. Unsurprisingly, given the association between variants in *FTO* and BMI [Frayling et al., 2007], there is strong evidence of heterogeneity in allelic odds ratios between obesity categories for SNPs in this gene (*P* = 7.2 × 10^−7^ at rs7193144). As a result, there is evidence of association with T2D obtained from the multinomial regression analysis (*P* = 9.2 × 10^−13^) is considerably more convincing than that from the logistic regression analysis of all cases combined (*P* = 2.8 × 10^−8^). Using the simulation procedure described above, we have estimated the power of the multinomial and logistic regression-based analyses, respectively, at a genome-wide significance level of *P*<5 × 10^−7^, to be 99.8 and 98.0% for the lead SNP in *TCF7L2*, and 99.3 and 77.1% for the lead SNP in *FTO*.

**Table II:** Regions of the genome demonstrating evidence of association with T2D (*P*<10^−5^) in a multinomial regression analysis of cases categorized according to obesity

					*P*-value			Allelic OR (95% confidence interval)	
						
Lead SNP	Chromosome	Location (Mb)	Gene (∼nearest)	Control MAF	Multinomial	Heterogeneity	Logistic	BMI≥30	BMI>30
rs4506565	10	114.75	*TCF7L2*	0.325	4.03 × 10^−14^	3.09 × 10^−4^	3.03 × 10^−12^	1.54 (1.38–1.72)	1.20 (1.08–1.34)
rs7193144	16	52.37	*FTO*	0.397	9.23 × 10^−13^	7.21 × 10^−7^	2.77 × 10^−8^	1.07 (0.96–1.19)	1.48 (1.33–1.64)
rs11176733	12	66.10	∼*CAND1*	0.063	2.20 × 10^−6^	2.90 × 10^−6^	4.10 × 10^−2^	0.84 (0.67–1.05)	1.51 (1.26–1.82)
rs7132840	12	69.70	∼*PTPRR*	0.457	4.65 × 10^−6^	1.21 × 10^−2^	1.93 × 10^−5^	1.30 (1.17–1.45)	1.11 (1.00–1.22)
rs901130	15	72.36	*CCDC33*	0.301	5.53 × 10^−6^	3.18 × 10^−4^	7.97 × 10^−4^	0.98 (0.87–1.10)	0.75 (0.67–0.84)
rs9465871	6	20.83	*CDKAL1*	0.178	5.61 × 10^−6^	5.86 × 10^−1^	1.02 × 10^−6^	1.32 (1.16–1.50)	1.26 (1.11–1.44)
rs387896	12	5.67	*TMEM16B*	0.106	8.70 × 10^−6^	1.22 × 10^−1^	4.80 × 10^−6^	0.78 (0.65–0.95)	0.65 (0.53–0.79)

Results are presented for the lead SNP in each region, including MAF in control samples, *P*-values for tests of association with T2D in multinomial and logistic regression frameworks, *P*-value for test of heterogeneity of odds ratios for T2D between obese and non-obese cases, and odds ratios (95% confidence intervals) for the minor allele in obese and non-obese cases.

Unlike previous studies, we have not investigated association of SNPs with BMI as a quantitative trait in cases of T2D [Frayling et al., 2007; Timpson et al., 2009]. If we were to do this, we would be focusing on the identification of variants contributing effects to obesity in T2D cases, as opposed to variants contributing effects to T2D, allowing for the possibility that these effects differ between obese and non-obese cases. The two tests are thus assessing the evidence against subtly different null hypotheses of no association.

Amongst the other signals of association with T2D identified through application of the multinomial regression model (Table II), two regions demonstrate clear evidence of heterogeneity in allelic odds ratios between obese and non-obese cases: variants close to *CAND1* (lead SNP rs11176733, with multinomial *P* = 2.2 × 10^−6^) and variants in *CCDC33* (lead SNP rs901130, with multinomial *P* = 5.5 × 10^−6^). Previous analysis of the WTCCC T2D cohort, stratified by obesity, identified a signal of association with variants in *CCDC33* in obese cases only [Timpson et al., 2009], the same effect as observed in our multinomial regression analysis. However, on follow in independent samples of UK origin [Zeggini et al., 2007], the association signal failed to replicate. More interestingly, the association of variants flanking *CAND1* with T2D has not been previously described. The association is limited to obese cases, demonstrating a similar pattern of heterogeneity in allelic odds ratios to those in *FTO*, and warrants follow-up in replication cohorts.

## DISCUSSION

We have developed a novel test of disease association with SNPs, allowing for heterogeneity in allelic odds ratios between sub-phenotypes, within a multinomial regression framework. This framework is extremely flexible, and can incorporate covariates to account for non-genetic risk factors and confounders, such as axes of genetic variation defining underlying population structure. Although we have presented results based on a multiplicative disease risk assumption within each sub-phenotype class, applied to directly observed genotypes, the multinomial regression model can easily be extended to incorporate more general disease models by incorporating dominance, and can be utilized with imputed data. Within the multinomial regression framework, we can also perform formal tests of heterogeneity in allelic odds ratios between sub-phenotypes.

The results of our simulation study highlight two general conclusions. First, the multinomial regression-based analysis performs well in comparison to existing methods formulated in a logistic regression framework over a range of models incorporating heterogeneity of genetic effects between sub-phenotypes. Second, when genetic effects are consistent across sub-phenotypes, the loss in power of the multinomial regression analysis is minimal. Given the multifarious genetic architecture underlying complex traits, it is not unreasonable to believe a model of heterogeneity of effects between sub-phenotypes. It is in this setting that the advantages of the multinomial regression approach will be maximized. Our results also highlight, in the context of two sub-phenotypes, that the multinomial regression-based test of heterogeneity has equivalent power to a direct comparison of the two case groups via logistic regression. However, the advantage of the multinomial regression approach is generalization to more than two sub-phenotypes in a unified analysis. In a logistic regression context, we would need to make comparisons between each pair of sub-phenotypes, making interpretation of heterogeneity statistics more complex, and would require correction for multiple testing.

We have applied the multinomial regression analysis method to a GWA study of T2D from the main WTCCC experiment [The Wellcome Trust Case Control Consortium, 2007], where cases were categorized cases according to obesity. Given the strong interplay between the two phenotypes, we expected that multinomial regression analysis might reveal additional T2D associations mediated through obesity and non-obesity related pathways. Our analysis provides: (i) stronger signals of association at established T2D loci than logistic regression-based methods applied to all cases combined; (ii) confirmation of previous findings of heterogeneity in genetic effects at *TCF7L2* and *FTO* according to obesity sub-phenotype in the same samples [Timpson et al., 2009]; and (iii) evidence of a novel potential T2D association with variants flanking Cullin-associated and neddylation-dissociated protein 1 *(CAND1)* in obese cases only. *CAND1* is a regulatory protein that interferes with the assembly of the SKP1-CUL1-F-box protein (SCF) ubiquitin ligase complex and thereby down-regulates ubiquitination of target proteins, and is involved in ubiquitin-dependent protein catabolic process. In a meta-analysis of three GWA studies of T2D [Zeggini et al., 2008], there was no evidence of association with SNPs in *CAND1* (*P* = 0.12). However, the meta-analysis did not focus on obese cases of T2D, and thus might not be expected to highlight the association we have identified through multinomial regression analysis of obesity sub-phenotypes. As a result, this signal warrants further follow-up in independent replication samples from the UK or closely related populations to confirm our findings, ideally focusing on obese cases of T2D, or by making use of multinomial regression to allow for heterogeneity of effects according to BMI.

We have presented the multinomial regression framework as a powerful approach to the analysis of sub-phenotypes. However, the utility of this method can be utilized in other genetic association contexts. For example, we could consider applying multinomial regression to comparisons of *related* phenotypes, such as CD and ulcerative colitis, against a combined control group. The multinomial regression approach will have greater power to detect pleiotropic loci, contributing the same, or different, effects to each phenotype, than would traditional analysis of each case-control cohort separately. Furthermore, by combining control cohorts, providing that they are suitably matched, we may also increase power to detect loci that contribute effects to just one phenotype.

The multinomial regression model assumes no ordering in the disease sub-phenotypes. However, for sub-phenotypes defined by severity, for example, we may wish to consider case categories as ordinal. In this setting, we can make use of the same statistical techniques as described above, but assume a proportional odds model for disease risk. This framework requires fewer parameters than the multinomial regression model, and thus may offer greater power to detect association if sub-phenotypes can be appropriately ordered.

It is clear that, for many complex traits, it is difficult to define one unified phenotype with the same underlying genetic risk factors, and that to do so may, in fact, be misleading. For example, cases of T2D may be affected as a result of beta cell failure and/or insulin resistance, and we might naturally expect that there are different genetic effects contributing to these two distinct pathways. With larger, and more clearly refined disease collections, our multinomial regression approach thus provides a powerful approach to detect variants contributing to the phenotype overall, whilst also highlighting those that may be specific to one category of disease. In this way, we can further our understanding of the biological mechanisms underlying disease, ultimately leading to improved, and more targeted therapies.
